# Supplementary material for: The fragmentation-induced fluidisation of pyroclastic density currents
Source: Nat Commun. 2023 Apr 12;14:2079. doi: 10.1038/s41467-023-37867-1 (PMC10097808; doi:10.1038/s41467-023-37867-1)
Supplement: Supplementary file 1 — Supplementary Information [file 41467_2023_37867_MOESM1_ESM.pdf]

Supplementary Information

The fragmentation-induced fluidisation of pyroclastic density currents

Eric C.P. Breard[1,2], Josef Dufek[2], Sylvain Charbonnier[3], Valentin Gueugneau[3], Thomas Giachetti[2], Braden Walsh[4]

[1] School of Geosciences, University of Edinburgh, Edinburgh, United Kingdom

[2] Department of Earth Sciences, University of Oregon, Eugene, OR, United States

[3] School of Geosciences, University of South Florida, Tampa, FL, United States

[4] Centre for Natural Hazards Research, Department of Earth Sciences, Simon Fraser University, Burnaby, BC, Canada

Corresponding Author: Eric.Breard@ed.ac.uk

Supplementary Note 1

While our simulations simplified the GSD of BAFs to a bidisperse distribution, our multiphase simulations enable us to calculate the elutriation flux for a representative case (Fig. S2, See Methods) and compare it to the empirical law proposed by [1].

$Q_e = a_3 U^3$ (Eq.1)

In their model, a_3 (in kg.s^2.m^{-5}) is a variable tuned to match natural deposit extent after eruptions. In our simulations, elutriation is largely driven by compaction due to the fragmentation of clasts. The flux predicted by our simulation agrees with values of Q_e determined at *posteriori* for BAFs at Merapi. In addition, based on the vertical gas-particle slip velocity, we calculated the maximum grain-size that would be fully coupled to fluid phase and elutriated out of the underflow. These results suggest particles under 50 microns in the first two kilometres and <30 microns beyond that distance would be transported out of the mixture, which could explain the depletion of fine ash (<32 microns) in the final deposits.

Boundary conditions<br>➢ Left/right side<br>➢ Top<br><br>➢ Bottom	 ➢ Periodic<br>➢ Outflow (constant atmospheric fluid pressure)<br>➢ Partial-slip
Initial conditions<br>➢ Fluid<br>➢ Temperature of the solid and gas mixture<br>➢ Grain-size at the start<br>➢ Solid density<br>➢ Internal friction coefficient<br>➢ Basal friction coefficient<br>➢ Restitution coefficient<br>➢ Pressure	 ➢ Air<br>➢ 773.15 K<br><br>➢ 0.01 m<br>➢ 2800 kg/m^3<br>➢ 0.7<br>➢ 0.7<br>➢ 0.5<br>➢ Hydrostatic (101325 Pa at the top of the domain)

Supplementary Table 1. Initial and boundary conditions of the MFIX simulations. These conditions were used in the multiphase flow modelling using the MFIX solver.

**Parameter changed**	**Impact on the maximum Pg***	**Runout**
3D instead of 2D with width = 5x flow height	+5%	+4%
Size segregation factor (changed from 0.3 to 0)	-0.1%	-0.2%
Cell vertical resolution /2	+0.4%	+12%
Cell vertical resolution x1.5	-0.02%	-0.05%
Initial temperature (-100 K)	-0.2%	-0.8%
Changed from Merapi to average volcanic slope	+2.4%	-2.5%
Basal boundary condition of solid (partial slip to no slip)	-9%	-0.3%

Supplementary Table 2. Sensitivity Analysis for the multiphase flow modelling. Changing individual initial and boundary condition parameters behind the simulations presented in Fig.3 and their impact on the maximum degree of fluidisation (Pg*) and final runout of the current.

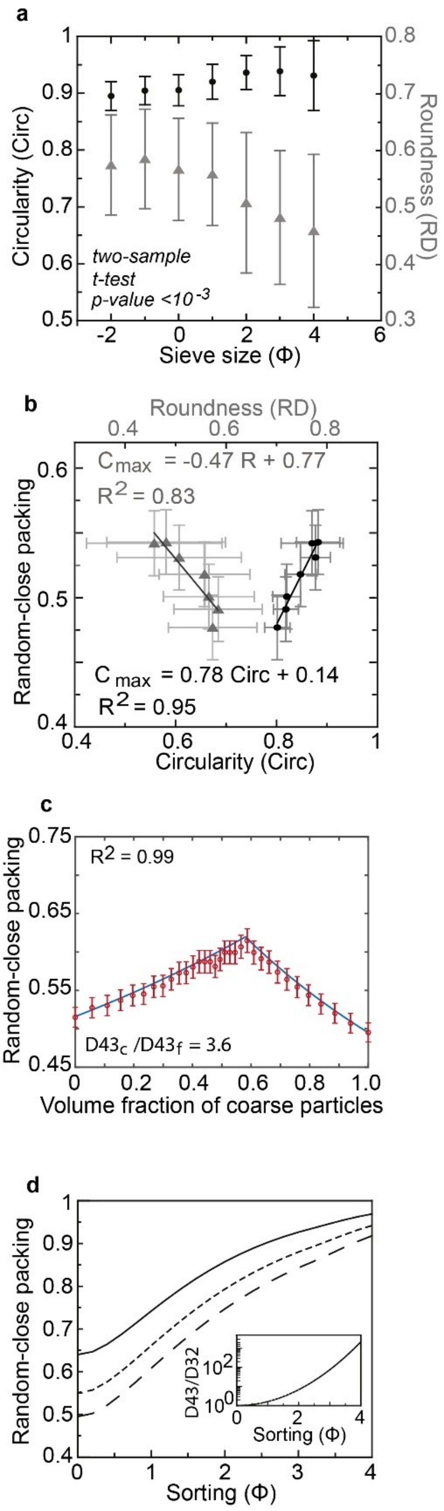

Supplementary Figure 1. Random-close packing and particle shapes in polydisperse volcanic mixtures. a, Roundness and circularity shape parameters for different particle size fraction of BAF material. **b**, Relationship between random-close packing and shape parameters for BAF material. **c**, Experimental measurements of random-close packing of BAF material with D43 size ratio between the coarse and fine fractions $D43_c/D43_f$ =3.6 and theoretical prediction based on the packing of the two endmembers using Yu and Standish [2]. **d**, Theoretical predictions of the random-close packing of all BAF samples for the three events at Merapi volcano, plotted against distance. Phi unit $\Phi=-\log_2(d[mm])$.

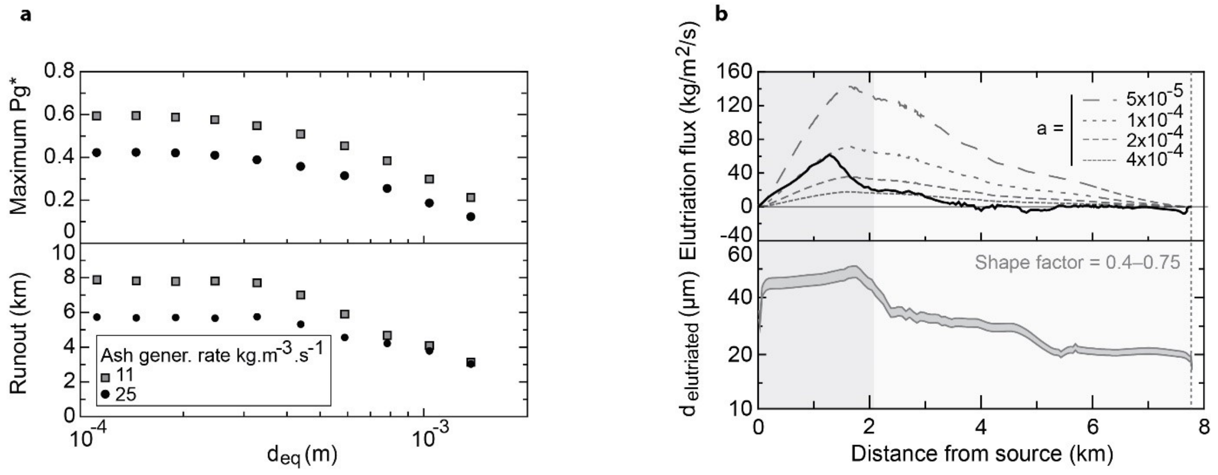

Supplementary Figure 2. Effect of mean diameter on the fragmentation-induced fluidisation and elutriation process. a, Maximum excess pore pressure and runout with respect to mean diameter (Sauter mean) of the binary mixture. **b**, Elutriation flux of the fine particles (100 microns) in MFIX simulation (black line) with initial conditions: H=18 m and ash generation rate =25 kg.m^{-3}.s^{-1}. The dotted lines represent the estimation based upon the empirical formula $Q_e = a_3 U^3$ where U is the depth-average flow velocity and "a_3" is a tuning parameter. The values presented as in line with that estimated a-posteriori by [1]. The bottom plot shows the largest particle diameter that can be elutriated by the upward gas flux in the dense mixture predicted by MFIX for the simulation with initial condition shown in the top plot. The data suggests fine ash was constantly elutriated during the propagation of the flow downslope rather than being increasingly stored within the basal avalanche, which explains the fine-depletion in the final deposit.

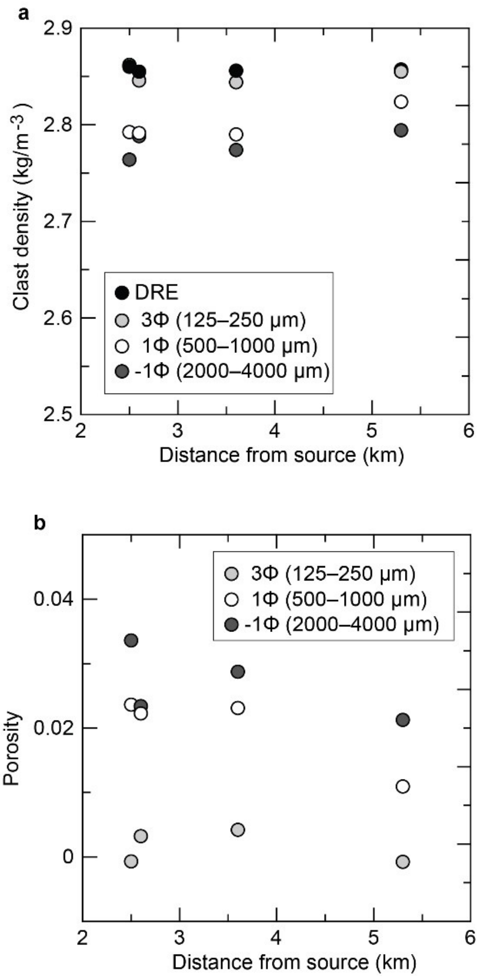

Supplementary Figure 3. Clast density and total porosity of the BAF material from the 26 October 2010 eruption. **a**, Clast density and for three sieved class fractions (-1, 1 and 3 phi) and dense rock equivalent (DRE) with distance from source. **b**, Total porosity of the clasts versus distance. The low porosity is commensurate with a flow initiation by gravitational collapse of the degassed dome.

Supplementary References

1 Kelfoun, K. A two-layer depth-averaged model for both the dilute and the concentrated parts of pyroclastic currents. *J. Geophys. Res. Solid Earth* **122**, 4293-4311, (2017).

2 Yu, A. B. & Standish, N. Estimation of the porosity of particle mixtures by a linear-mixture packing model. *Industrial & Engineering Chemistry Research* **30**, 1372-1385, (1991).
