# Supplementary material for: The fragmentation-induced fluidisation of pyroclastic density currents
Source: Nat Commun. 2023 Apr 12;14:2079. doi: 10.1038/s41467-023-37867-1 (PMC10097808; doi:10.1038/s41467-023-37867-1)
Supplement: Supplementary file 3 — Description of Additional Supplementary Files [file 41467_2023_37867_MOESM3_ESM.pdf]

File name: Supplementary Movie 1
Description: Video of the PDC simulation output created using the VolcFlow depth-average model with initial conditions S1 (Figure 4).

File name: Supplementary Movie 2
Description: Video of the PDC simulation output created using the VolcFlow depth-average model with initial conditions S1 (Figure 2).

File name: Supplementary Movie 3
Description: Video of the PDC simulation output created using the VolcFlow depth-average model with initial conditions S3 (Figure 4).

File name: Supplementary Movie 4
Description: Video of the PDC simulation output created using the VolcFlow depth-average model with initial conditions S4 (Figure 4).

File name: Supplementary Movie 5
Description: Video of the PDC simulation output created using the VolcFlow depth-average model with initial conditions S5 (Figure 4).

File name: Supplementary Movie 6
Description: Video of the PDC simulation output created using the VolcFlow depth-average model with initial conditions S6 (Figure 4).

File name: Supplementary Movie 7
Description: Video of the PDC simulation output created using the VolcFlow depth-average model with initial conditions S7 (Figure 4).

File name: Supplementary Movie 8
Description: Video of the PDC simulation output created using the VolcFlow depth-average model with initial conditions S8 (Figure 4).

File name: Supplementary Movie 9
Description: Video of the PDC simulation output created using the VolcFlow depth-average model with initial conditions S9 (Figure 4).

File name: Supplementary Movie 10
Description: Video of the PDC simulation output created using the VolcFlow depth-average model with initial conditions S10 (Figure 4).

File name: Supplementary Movie 11
Description: Video of the PDC simulation output created using the VolcFlow depth-average model with initial conditions S11 (Figure 4).

File name: Supplementary Movie 12
Description: Video of the PDC simulation output created using the VolcFlow depth-average model with initial conditions S12 (Figure 4).

File name: Supplementary Movie 13
Description: Video of the PDC simulation output created using the VolcFlow depth-average model with initial conditions S13 (Figure 4).
